# Auxin efflux carrier PsPIN4 identified through genome-wide analysis as vital factor of petal abscission

**DOI:** 10.3389/fpls.2024.1380417

**Published:** 2024-05-10

**Authors:** Yin Sun, Junqiang Chen, Yanchao Yuan, Nannan Jiang, Chunying Liu, Yuxi Zhang, Xiuhong Mao, Qian Zhang, Yifu Fang, Zhenyuan Sun, Shupeng Gai

**Affiliations:** ^1^ State Key Laboratory of Efficient Production of Forest Resources, Key Laboratory of Tree Breeding and Cultivation of the National Forestry and Grassland Administration, Research Institute of Forestry, Chinese Academy of Forestry, Beijing, China; ^2^ Shandong Provincial Key Laboratory of Forest Genetic Improvement, Yellow River delta forest ecosystem positioning research station, Shandong Provincial Academy of Forestry, Jinan, China; ^3^ University Key Laboratory of Plant Biotechnology in Shandong Province, College of Life Sciences, Qingdao Agricultural University, Qingdao, China

**Keywords:** auxin, PsPINs, tree peony, phosphorylation, abscission

## Abstract

PIN-FORMED (PIN) proteins, which function as efflux transporters, play many crucial roles in the polar transportation of auxin within plants. In this study, the exogenous applications of auxin IAA and TIBA were found to significantly prolong and shorten the florescence of tree peony (*Paeonia suffruticosa* Andr.) flowers. This finding suggests that auxin has some regulatory influence in petal senescence and abscission. Further analysis revealed a total of 8 *PsPINs* distributed across three chromosomes, which could be categorized into two classes based on phylogenetic and structural analysis. PsPIN1, PsPIN2a-b, and PsPIN4 were separated into the “long” PIN category, while PsPIN5, PsPIN6a-b, and PsPIN8 belonged to the “short” one. Additionally, the cis-regulatory elements of *PsPIN* promoters were associated with plant development, phytohormones, and environmental stress. These genes displayed tissue-specific expression, and phosphorylation sites were abundant throughout the protein family. Notably, *PsPIN4* displayed distinct and elevated expression levels in roots, leaves, and flower organs. Expression patterns among the abscission zone (AZ) and adjacent areas during various flowering stages and IAA treatment indicate that *PsPIN4* likely influences the initiation of peony petal abscission. The PsPIN4 protein was observed to be co-localized on both the plasma membrane and the cell nucleus. The ectopic expression of *PsPIN4* reversed the premature flower organs abscission in the *Atpin4* and significantly protracted florescence when introduced to Col *Arabidopsis*. Our findings established a strong basis for further investigation of *PIN* gene biological functions, particularly concerning intrinsic relationship between PIN-mediated auxin polar

## 1 Introduction

Auxin is a ubiquitous plant hormone that establishes an intertissue concentration gradient through specialized polar transport to serve various crucial roles in the regulation of plant growth, development, and response to external cues (Enders and Strader, 2015). Auxin transporters can be divided into two groups based on function and location. The first group primarily facilitates intercellular auxin transportation and comprises transporters located on the plasma membrane such as AUXIN1/LIKE AUX1 (AUX/LAX), pin-formed (PIN), and ATP-binding cassette (ABC). The second group features transporters that regulate intracellular auxin homeostasis and are distributed within intracellular compartments, including several PINs with short hydrophilic loops, PIN-Like transporters (PILS), and WALLS ARE THIN 1 (WAT1) (Ranocha et al., 2013; Kuhn et al., 2017; Zhou and Luo, 2018).

The PIN protein family is expansive, with diverse members identified in over 30 plant species (Yue et al., 2015; Huang et al., 2020). Driven by evolution, sub-functionalization or new functionalization has led to significant modifications in protein structure (Mravec et al., 2009; Ditengou et al., 2018). Members of this family are crucial auxin transporters, making them vital components of various auxin-regulated processes such as organogenesis, development, tropism, and morphogenesis. To ensure precise auxin delivery, PIN proteins must be strategically distributed within a plant. Categorized as short or long, these proteins inhabit separate cellular spaces and serve distinct roles. Typically, long PIN proteins are polarized on the cell membrane to facilitate intercellular auxin transport. For example, auxin circulation in root tips is facilitated by the distinct polar localization patterns of *Arabidopsis* PIN1-3, PIN4, and PIN7 (Blilou et al., 2005; Paponov et al., 2005; Vieten et al., 2005).Additionally, during embryonic development, the PIN1 and PIN7 proteins orient themselves toward the suspensor to direct auxin flow toward the structure (Friml et al., 2003; Weijers et al., 2005). In tomatoes (*Solanum lycopersicum*), *SlPIN1* heavily influence leaf development and formation of leaf clefts (Koenig et al., 2009). *SlPIN4* is highly expressed in tomato flowers and fruit, particularly in the placenta during fruit development (Mounet et al., 2012; Pattison and Catala, 2012). The “short” PIN is primarily localized in the endoplasmic reticulum, where they regulate intracellular auxin homeostasis. In *Arabidopsis*, auxin transport is primarily overseen by PIN5 and PIN8. PIN5 is predominantly expressed in hypocotyls, cotyledon vascular tissue, and guard cells, while PIN8 exhibits specific expression in anthers, male gametophytes, and sporophytes (Mravec et al., 2009; Barbez et al., 2012; Ding et al., 2012). Moreover, PIN6 displays dual localization and can be observed on both the cell membrane and endoplasmic reticulum (Bender et al., 2013; Cazzonelli et al., 2013; Simon et al., 2016).

PIN protein activity and distribution are finely regulated by plant hormones (Vieten et al., 2005; Ruzicka et al., 2007). With few exceptions, including *PIN5*, most *PINs* are mediated by a positive feedback mechanism within the Aux/IAA-ARF signal transduction pathway (Gallavotti et al., 2008; Bayer et al., 2009; Mravec et al., 2009). For example, the expression and polarity of *PIN1* are determined by an intricate yet elusive system involving *ARF5* and *IAA12* (Wenzel et al., 2007; Garrett et al., 2012). In *Arabidopsis*, gravitropic set-point angles of lateral roots are controlled by the cooperative action of MYB88 and the R2R3-MYB transcription factor FOUR LIPS (FLP, MYB124), which target the promoters of *PIN3* and *PIN7* (Wang et al., 2015). Finally, the transcription factor, WRKY23 acts downstream of the auxin signaling pathway to mediate feedback on the polarized auxin transport (Prát et al., 2018). Additionally, PINs are regulated by a complex functional network consisting of various hormones such as ethylene (ETH), cytokinin (CTK), fatty acid, brassinolide (BL), jasmonic acid (JA) (Li et al., 2005; Ruzicka et al., 2007, Ruzicka et al., 2009; Peer et al., 2013; Jang et al., 2019). Multiple To function correctly, PIN proteins must be phosphorylated at multiple conserved sites along the hydrophilic loop, which serve as specific targets for kinases (Weller et al., 2017; Barbosa et al., 2018). Among these sites in *Arabidopsis*, serine residues S1-S3 (S231, S252, and S290) within three highly conserved TPRXS motifs are targeted by PIDs (PINOID) and D6 protein kinases (D6PKs). In addition, MKK7-MAPK3/6 cascades phosphorylated S337 to depolarize the hypocotyl and stem (Zourelidou et al., 2014; Dory et al., 2018; Hajny et al., 2020).

Abscission represents a crucial developmental stage during which organs are separated from plant bodies in a region known as the abscission zone (AZ), which exhibits a high sensitivity towards shedding signals (Tucker and Yang, 2012; Wilmowicz et al., 2018; Shi et al., 2023). The timing of organ detachment is governed by plant hormones such as ethylene, auxin, and abscisic acid (ABA) signaling the activation of various Metabolic processes (Dal Cin et al., 2007; Sun, 2009; Meir et al., 2010; Parra-Lobato and Gomez-Jimenez, 2011; Tadeo et al., 2012a). Early abscission is prevented by auxin synthesized in distal organs and transported through PIN proteins, which inhibit the responses to ethylene signaling (Zhu et al., 2011; Kühn et al., 2016). Once a reduction in auxin is detected in the AZ, cells become responsive to these signals and trigger abscission (Meir et al., 2006). During rose (*Rosa hybrida*) petal abscission, there is a reduction in the amount of auxin and *RhSUC2* from the phloem of AZ to the petals. This auxin influences the shedding process by binding to response factor *RhARF7*, which then regulates the *RhSUC2* promoter (Liang et al., 2020). Furthermore, previous research investigating tomato pedicel abscission has characterized the regulatory roles of *SlPIN1* and *SlPIN4*, which facilitate the basal transportation of auxin to the AZ and promote pedicel abscission (Shi et al., 2017; Liang et al., 2020).

The tree peony (*Paeonia suffruticosa* Andr.) is a woody flowering plant that has long been cultivated for its vibrant colors, elegant posture, and expansive floral patterns. However, the species’ ornamental and economic value is hindered by a short flowering period, with plants often blooming for less than one week under natural conditions. Although a growing body of research supports an association between auxin and abscission, few studies have explored the role of PIN-mediated auxin transport proteins in this process. In this study, we comprehensively investigate the *PIN* gene family by using genome sequence data from tree peony to analyze gene structures, chromosomal localization, phylogenetic relationships, motif, transmembrane structures, and cis-elements. Additionally, we analyzed gene expression profiles in various tissues to speculate on their potential functions while striving to identify members involved in initiating AZ formation. Our findings lay a foundation for further functional *PsPIN* investigations and explore the potential connections between PINs, auxin signaling pathways, and plant organ abscission.

## 2 Materials and methods

### 2.1 Plant materials and treatments

A total of 30 tree peonies (cv. ‘Xueyingtaohua’) were planted and grown for four years in a greenhouse at Qingdao Agricultural University (Qingdao, China) (22 ± 3 °C day, 15 ± 3 °C night, natural 10 h light/14 h dark photoperiod). Five slightly opening flowers in similar bud states were selected and removed from each test plant. Samples were delivered to the laboratory within one hour of harvest and stems were pruned to 15 cm, immersed in distilled water, and placed in an incubator (20 °C, 14 h light/10 h dark) for 36 hours. Twenty cut flowers were randomly selected from each group and treated with either a solution containing 100 μM, 200 μM IAA (Indole-3-Acetic Acid, IAA) and 100 μM TIBA (2,3,5-triiodobenzoic acid, TIBA). The remaining samples were still placed in distilled water as the control group. 10 cut flowers were randomly selected from each treatment group and control group for shedding rate statistics. Following treatment, organs of interest were individually removed from the flowers. Double-sided blades were used to excise the ovary by cutting along its boundary with the receptacle. Whole stamens were removed and kept intact. The abscission zone was represented by the 2 mm segment located at the junction between the petals and the receptacle. The proximal and distal portions of this section were represented by the upper 2 mm (including petal tissue) and lower 1mm (including the ring-shaped receptacle), respectively. Three cut flowers were selected for each sampling stage for *in situ* hybridization, fluorescence quantification, and endogenous IAA determination experiments, respectively.

### 2.2 Sequence retrieval and phylogenetic analysis

The known PIN protein sequences of *Arabidopsis thaliana* [TAIR - Home Page (arabidopsis.org)] and *Oryza sativa* [Rice Genome Annotation Project (uga.edu)]were downloaded to blast tree peony genomewide (Yuan et al., 2022). The HMMER software was utilized with default parameters to conduct a search for candidate PIN protein sequences according to transmembrane domain (PF03547.22), followed by further identification of the sequences through a homology search using the BLAST default parameters. The candidate tree peony *PIN* genes were validated using the SMART web tool [SMART: Main page (embl-heidelberg.de)], and manually curated to remove truncated and redundant proteins. The identified tree peony PIN proteins were designated based on their phylogenetic relationships with *Arabidopsis* homologs. The subcellular localization information of the PsPIN protein sequences were analyzed by the Softberry service platform ProtComp 9.0 (SoftBerry - ProtComp (Plant) HELP) (Yu et al., 2008a). The MapInspect software (http://mapinspect.software.informer.com/) (Wu et al., 2019) was used to analyze physical position and chromosomal localization of *PsPIN* genes. The MEME web server [Introduction - MEME Suite (meme-suite.org)] (Bailey et al., 2015) was employed to identify intricate motifs of PsPIN proteins, utilizing the default parameter settings except for a maximum motif count of 20. A total of 112 PIN amino acid sequences from 11 species (*Arabidopsis thaliana*, *Paeonia ostii*, *Solanum lycopersicum*, *Solanum tuberosum*, *Gossypium raimondii*, *Zea may*, *Oryza sativa*, *Cystopteris fragilis*, *Picea abies*, *Populus trichocarpa*, and *Glycine max*) were retrieved from the NCBI database. Multiple sequence alignment was performed using ClustalW program in MEGA-X software (Kumar et al., 2018), and the phylogenetic tree was constructed using maximum likelihood (ML) with 1000 bootstraps.

### 2.3 Gene structure, cis-acting elements and phosphorylation sites analysis

The *PsPIN* gene structure (exon-intron) was defined using the online software Gene Structure Display Server (GSDS) [Gene Structure Display Server 2.0 (gao-lab.org)] (Hu et al., 2015). DNAMAN software (https://www.lynnon.com/pc/framepc.html) and TMHMM Serverv.2.0 (http://www.cbs.dtu.dk/services/TMHMM/) were applied to perform protein family sequence alignment and transmembrane structure analysis. The *cis*-regulatory elements in the promoter region (2,000 bp upstream of the starting codon) of the *PsPINs* were searched by the online program of PlantCARE [PlantCARE, a database of plant promoters and their cis-acting regulatory elements (ugent.be)] (Lescot et al., 2002). Gene structure, cis-acting element numbers and responsive functions were visualized using TBtools (Chen et al., 2020). NetPhos 3.1 web server (NetPhos 3.1 - DTU Health Tech - Bioinformatic Services) was used to analyze the phosphorylation sites of the PsPIN proteins.

### 2.4 Real-time quantitative RT-PCR

Total RNA was extracted from 100 mg of each sample using a Steady Pure Plant RNA Extraction Kit (TaKaRa, Beijing, China) according to the manufacturer’s instructions. The first strand of cDNA was synthesized from 1 µg of total RNA using a HiScript III RT SuperMix (Vazyme, Nanjing, China) for qPCR reverse transcriptase at a final volume of 50 µL. qRT-PCR was performed using a SYBR^®^ Premix Ex Taq™II Kit (TaKaRa, Dalian, China). All reactions were performed in triplicate. The relative expression level was calculated by the 2^-ΔΔCt^ method (Livak and Schmittgen, 2001). The primers used for qRT-PCR are listed **Supplementary Table S8**.

### 2.5 IAA quantifcation by ELISA

IAA standards, antigens, and antibodies were obtained from Qingdao Agricultural University. All samples were homogenized and extracted in cold 80% methanol (4°C) containing 1mmol/L butylated hydroxytoluene for 4 h. Samples were then centrifuged at 4000rpm for 15 min (4°C). Extracts were passed through a C18 Sep-Pak cartridge (Waters, USA), dried using N_2_, and then dissolved in a mixture comprising 2 mL PBS (pH 7.5), 0.1% Tween 20 (v/v), and 0.1% gelatine (w/v). Next, 96-well microfiltration plates were coated with an IAA antigen in NaHCO_3_ buffer (50 mmol/L, pH 9.6) and incubated overnight at 4°C. Plates were then washed four times with a mixture of PBS (pH 7.5) and 0.1% Tween 20 (v/v). Subsequently, all wells were filled with 50 μL of IAA antibodies along with a 50 μL aliquot of either a sample extract or IAA standard. Plates were then incubated at 37°C for 30 min. After an additional wash with the PBS and Tween 20 mixture, wells were supplemented with IgG horseradish peroxidase, incubated for 30min at 37°C, and washed a final time. Finally, ortho-phenylenediamine was supplied to act as a substrate before a final 30 min incubation at 37°C. Absorbance was detected at 490 nm.

### 2.6 *In situ* hybridization

The AZ samples were collected at 0, 24, 48, and 72 hours after flowering for this assay. The *in vitro* transcription vector pGEM-T was constructed using a *PsPIN4*-specific fragment amplified. with the Antisense probe sequence is listed in **Supplementary Table S9**. Needle labeling was performed following the protocols provided by the DIG RNA Labeling Kit (SP6/T7) (Roche, USA) and DIG Nucleic Acid Detection Kit (Roche, USA). The probe concentrations used was 211.7 ng/ul for PIN. The protocol for *in situ* hybridization was modified based on Abcam’s guidelines ( In situ hybridization (ISH) protocol | Abcam). Detailed steps for paraffin sectioning referred to Shi (Shi et al., 2017), finally, the sections were stained overnight with NBT/BCIP (Roche, Germany) at 37°C. Subsequently, the samples were examined under a Nikon Eclipse ci optical microscope (Nikon, Japan).

### 2.7 Generation of transgenic plants and floral organs abscission assay

The overexpression vector *35S:PsPIN4* was generated by cloning the CDS of *PsPIN4* into the vector pCAMBIA1300. The primers used for *PsPIN4* CDS amplification are listed in **Supplementary Table S8**. Transgenic *Arabidopsis* plants were obtained according to the floral dip transformation method (Clough and Bent, 1998) and were then used for phenotype observation and further assays. The pBS was quantified as the force in gram equivalents required for pulling a petal from flower (Butenko et al., 2003), as determined by using a digital force gauge (Model: HF-2, Lunjie Electromechanical Instrument Co., Ltd., Shanghai). A total of 20 petals per position were measured.

### 2.8 Histochemical GUS assays

A promoter sequence of 2,000 bp upstream of the starting codon was cloned and fused into pCAMBIA1381 vector with a GUS reporter gene. The primers used for *PsPIN4* promoter amplification are listed in **Supplementary Table S8**. *Arabidopsis* homozygous T_3_ generation plants were utilized for GUS staining analysis. Samples at different stages (1, 2, 3, and 4 weeks) as well as floral organs of different positions (P1-P7) were selected to observe the expression of *ProPsPIN4* throughout the whole plant using GUS staining. The samples were performed in the staining solution (50 mM phosphate buffer (pH7.2), 0.1% (v/v) Triton ™ X-100, 0.5 mM K_4_Fe (CN) 6H_2_O, 0.5 mM K_3_Fe (CN) _6_, and 0.5 mM X-Gluc) at 37°C in the dark. Then 95% ethanol was applied to decolorize the tissues and examined under the ZEISSV11 stereoscope (Nikon Japan).

### 2.9 Western blot analyses

For immunoblot analyses, total proteins were extracted from 0.1 g of four transgenic lines (Arabidopsis thaliana at 1,2,3, and 4 weeks of age and floral organs at 2,4, and 6 positions) and NT plants in 3 ml of extraction buffer [50 mM Tris–HCl (pH 8), 10 mM MgCl_2_, 1 mM EDTA 0.5 M (pH 8), 5% glycerol, 1 mM DTT, 0.1% Triton X-100 and 1 mM (PMSF)]. The homogenate was placed on ice for 5 min, and then centrifuged at 13,000 *× g* for 15 min. The proteins concentrations of the resulting supernatant were determined by the Bradford method (Bradford, 1976). Total proteins samples were then electrophoretically separated on 12% (w/v) SDS–PAGE gels and blotted by the semi-dry method (iBlotTM Gel Transfer Stack) onto a nitrocellulose membrane (Invitrogen). GUS protein detection was carried by the same protocol described by Ben Saad et al. (Ben-Saad et al., 2015).

### 2.10 Subcellular localization of *PsPIN4*

The *PsPIN4* ORF without stop codon was amplified with the primers PsPIN4-GFPF and PsPIN4-GFPR (**Supplementary Table S8**). The PsPIN4::GFP fusion expression vector was constructed and used to transform Agrobacterium (*Agrobacterium tumefaciens*) GV3101. Agrobacterium was cultured in LB liquid medium (with kanamycin and rifampicin) until an OD_600_ of 0.6–0.8 was obtained. Further, the cells were centrifuged at 5,000 rpm for 10 minutes. The cell pellet was diluted to obtain an OD_600_ of 0.6–0.8 with a solution of 10 mM MES, 200 mM acetylsyringone, and 10 mM MgCl_2_ (pH 5.6), and injected into tobacco leaves. The infected plants were cultured in darkness for 24 hours, and then in normal conditions (25°C, 16 hours light/8 hours dark) for 24 hours. The nuclear labeled protein GHD7::CFP and the membrane labeled protein PIP2;1::RFP were interchanged for accurate localization, and the fluorescence was observed using a laser confocal microscope.

### 2.11 Statistical analysis

Statistical analysis was performed by GraphPad Prism 7 (GraphPad Software Inc. San Diego, CA. USA) and t-tests with P < 0.05 (∗) and P < 0.01 (∗∗) was used to indicate statistically significant level. All results were presented as the means ± stand*ard devi*ation (SD) and at least three replicates were set up.

## 3 Results

### 3.1 Identification of PIN proteins in tree peony

In this study, a total of 8 *PsPIN* genes were identified in tree peony and were renamed *PsPIN1*, *PsPIN2a-b*, *PsPIN4*, *PsPIN5*, *PsPIN6a-b* and *PsPIN8* (**Table 1**) according to *Arabidopsis* homologs. Information regarding these genes and their corresponding proteins including gene ID, location, number of exons, protein length (aa), molecular weight (MW), theoretical isoelectric point (pI), and subcellular location are showed in **Table 1** and **Supplementary Table S1**. Among the 8 PsPIN proteins, the lengths and molecular weights varied greatly, ranging from 90 aa (PsPIN6b) to 654 aa (PsPIN4) and from 10192.01 Da to 70955.96 Da, respectively. Our prediction of subcellular localization revealed that PsPIN proteins are primarily distributed on the cell membrane. In addition, the presence of shorter protein sequences may be attributed to the genome annotation process.

**Table 1:** The PIN family genes in tree peony.

Gene Name	Gene ID	Location	Exon	Protein length(aa)	MW(Da)	pI	Subcellular location
PsPIN1	Pos.gene80617	Chr01	6	602	64849.97	8.94	Cytoplasm
PsPIN2a	Pos.gene7433	Chr01	6	540	59431.93	9.08	Cell membrane
PsPIN2b	Pos.gene48530	Chr05	7	179	20088.07	5.1	Chloroplast
PsPIN4	Pos.gene23763	Chr05	6	654	70955.96	6.71	Cell membrane
PsPIN5	Pos.gene23140	Chr01	5	356	39175.29	6.64	Cell membrane
PsPIN6a	Pos.gene11355	Chr04	7	161	18401.57	8.91	Cell membrane
PsPIN6b	Pos.gene23815	Chr05	4	90	10192.01	5.99	Cell membrane
PsPIN8	Pos.gene47573	Chr04	5	357	39044.95	9.52	Cell membrane

**Figure 1:**
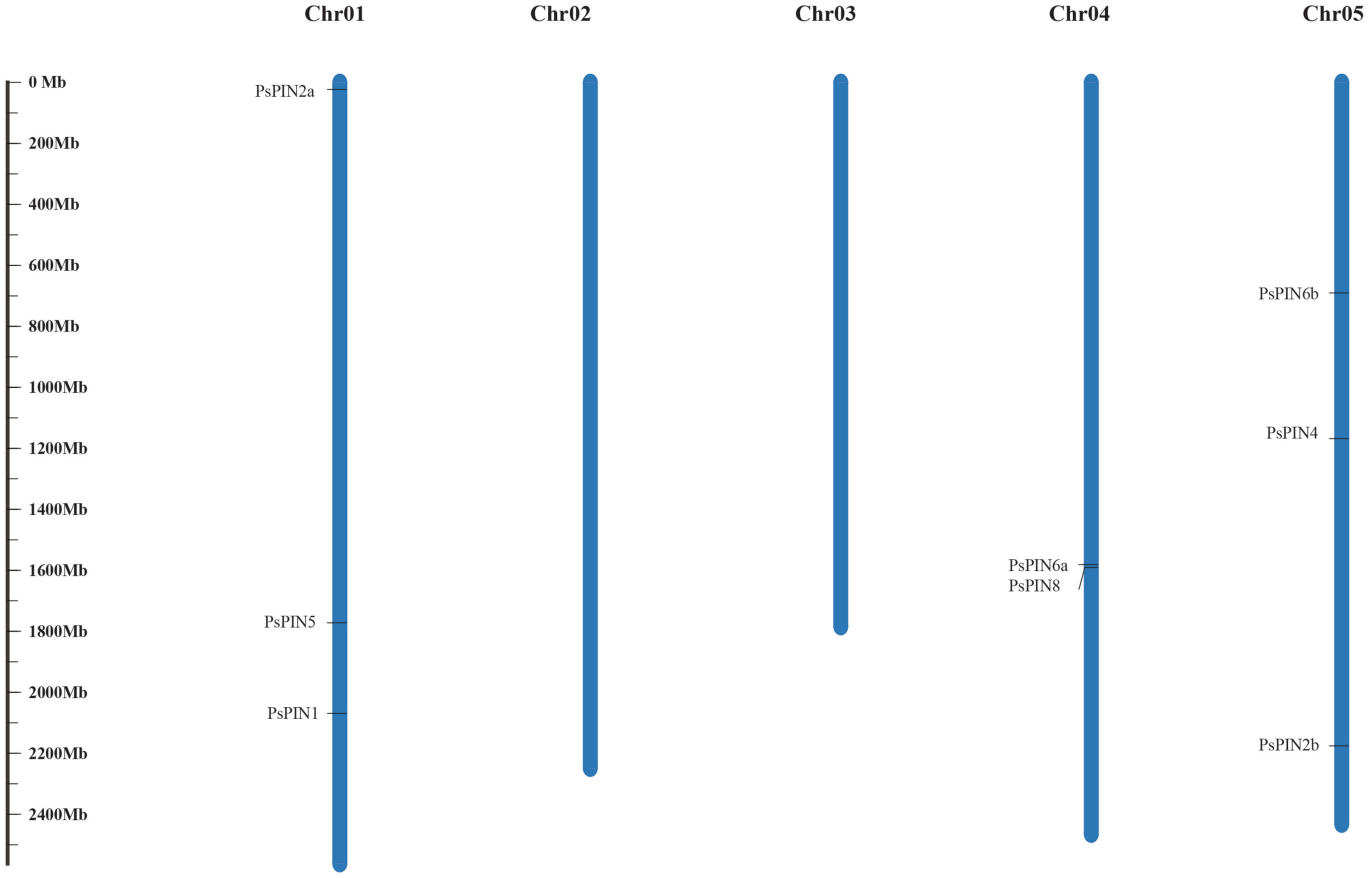
Distribution of *PsPIN* genes in tree peony. Scale bar indicates length of chromosome.

Through comparisons with the tree peony genome, our results indicate that the newly identified *PsPIN* genes are distributed across three chromosomes (**Figure 1**), the majority of which were located on chromosomes I and V (each containing three *PsPIN* members). In view of this, we searched the tree peony genome for segmental and tandem duplicate gene pairs and found no repeat events among *PsPIN* family members.

### 3.2 Multiple sequence alignment, phylogenetic analysis and motif assessment of PsPINs

**Figure 2:**
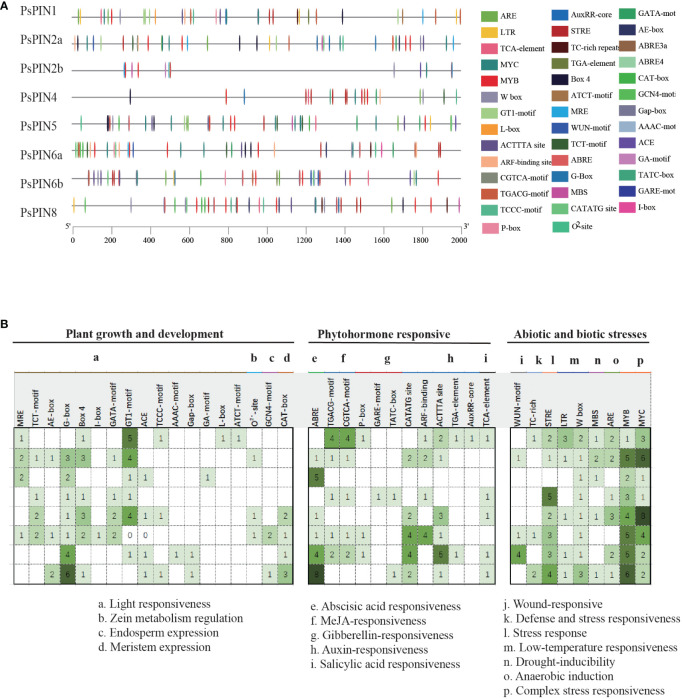
Evolution and Conserved motifs with PIN members **(A)** Phylogenetic tree of 112 PIN amino acid sequences collected from 11 species. PsPIN are labeled with red color (f2 s/b f4At; *Arabidopsis thaliana*; Ps, *Paeonia suffroticosa*; Sl, *Solanum lycopersicum*; St, *Solanum tuberosum*; Gr, *Gossypium raimondii*; Zm, *Zea may*; Os, *Oryza sativa*; Cf, *Cystopteris fragilis*; Pa, *Picea abies*; Pt, *Populus trichocarpa*; Gm, *Glycine max*). All proteins are listed in **Supplementary Table S2**. **(B)** Conserved PsPIN protein motifs in tree peony. Motifs are differentiated with colored boxes and numbers (1–20). Gray lines represent the non-conserved sequences. Sequence information for all motifs is provided in **Supplementary Table S3**.

To elucidate the evolutionary relationship within the *PsPIN* gene family, we used the MEGA-X software to construct a phylogenetic tree from the inferred full-length amino acid sequences of 112 PIN protein sequences collected from 11 species. Based on the classification methods of the AtPINs, the tree revealed six distinct branches (AtPIN1, AtPIN2, AtPIN5, AtPIN6, AtPIN8, and AtPIN3/4/7), The 8 newly identified PsPIN proteins were categorized into six groups: PsPIN1, PsPIN2a-b, PsPIN4, PsPIN5, PsPIN6a-b and PsPIN8. The evolutionary analysis revealed a robust correlation between peony and poplar PIN. We employed the MEME (Multiple Em for Motif Elicitation) to unveil the motif compositions of the 112 PIN proteins and identified 20 conserved motifs through structural analysis (**Figure 2**). In the “long” PIN group, PsPIN1 lacked only the 17th motif, whereas PsPIN2a lacked motifs 9, 12, 17, 18 and 20. Potentially as a result of incomplete genome annotation, only one or two motifs were identified in PsPIN2b, PsPIN6a, and PsPIN6b. Conversely, “short” PINs such as PsPIN5 and PsPIN8 were found to contain shorter central hydrophilic rings and lack the typical 10, 15, 11, 9, 18, 17, 12 and 14 motifs.

### 3.3 Structure and transmembrane domains analysis of the PsPINs

**Figure 3:**
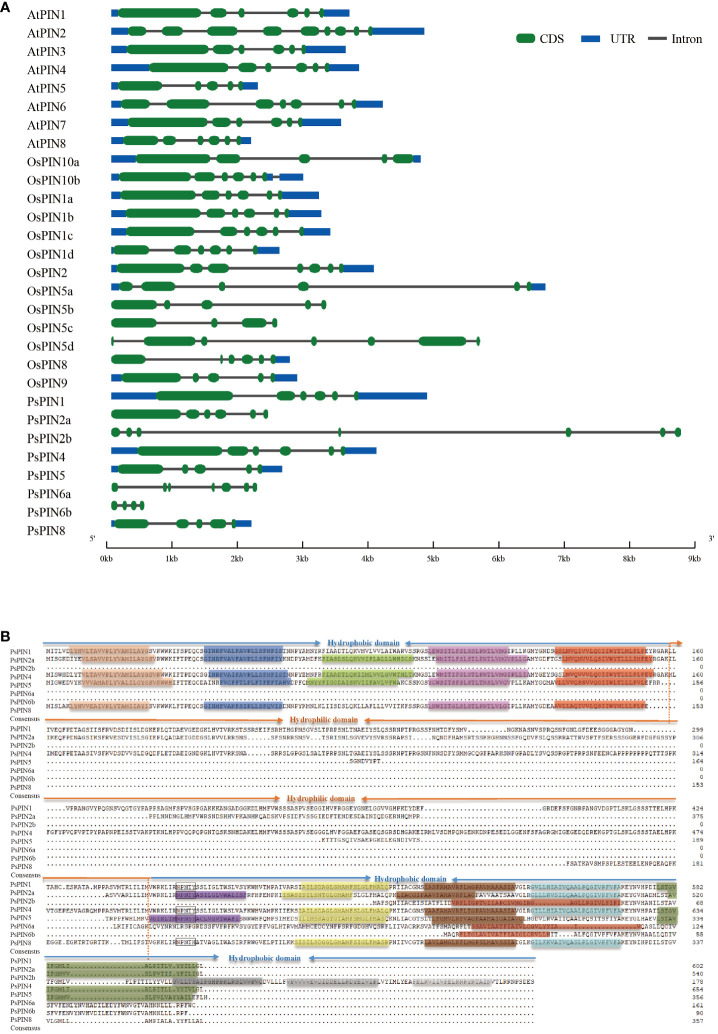
The structure and transmembrane domain analysis of PsPINs. **(A)** Intron-exon structure of *PsPINs*. Green boxes represent exons, blue boxes represent UTR, and spaces between the boxes represent introns. The scale bar indicate exon and intron length. **(B)** The transmembrane and modular loop structure of PsPINs. Prediction of trans-membrane helices within two highly conversed hydrophobic domains located at N- and C-termini are indicated by colored rectangles.

The structural characteristics of the gene family were investigated by integrating the *PIN* genes from *Arabidopsis*, rice, and tree peony. The results illustrated great variability, with the quantity of introns among the *PsPINs* ranging from 3 to 6 (**Figure 3A**). Specifically, *PsPIN5* and *PsPIN8* each contained four introns, while *PsPIN1*, *PsPIN2a*, and *PsPIN4* contained five introns, and *PsPIN2b* and *PsPIN6a* contained six introns. Due to potentially incomplete genomic annotation, only three introns were detected in *PsPIN6b*. It is important to highlight the absence of UTR regions associated with *PsPIN2* and *PsPIN6*, which also displayed shorter exon segments. Additionally, *PsPIN2b* possessed notably longer gene spans.

The transmembrane motif of the PsPIN protein was presented for further structural analysis (**Figure 3B**). We identified a distinct central hydrophilic ring (HL), which separates the two hydrophobic domains (HD) located at the N-terminus and C-terminus. The variances in length recorded in the central hydrophilic region contribute greatly to PsPIN diversity.PsPIN2a, PsPIN4, and PsPIN5 possessed five transmembrane helices (Helix1-Helix5) at the N-terminus, whereas PsPIN1 and PsPIN8 lacked the third transmembrane helix. Specifically, only PsPIN2a contained five transmembrane helices at the C-terminus, while PsPIN1 and PsPIN4 had 4 helices, and PsPIN8 had only 3 helices. The eighth helix of PsPIN2b, PsPIN6a, and PsPIN6b differed significantly from other members. The conserved NPXXY structure, which is crucial membrane and receptor proteins interaction in endocytosis, was found near the hydrophobic and hydrophilic regions of the C-terminus. Notably, the tyrosine (Y) in PsPIN8 was replaced with histidine (H). Amino acid fragments are shown in **Supplementary Table S4**.

### 3.4 Cis-elements and phosphorylation sites analysis of the *PsPIN* promoters

**Figure 4:**
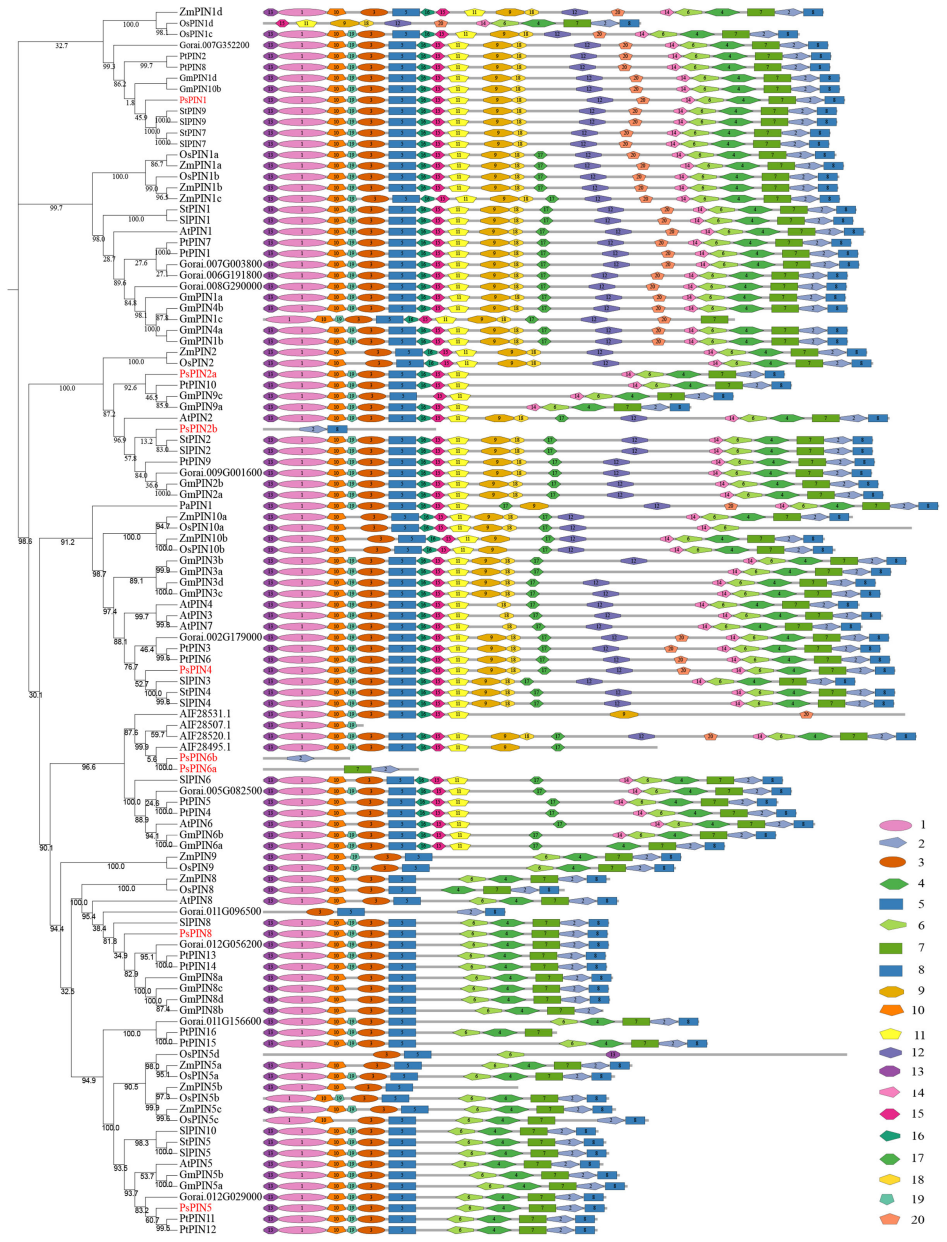
Predicted *cis-*elements in *PsPINs* promoters. *Cis*-regulatory elements in *PsPINs* promoter regions (2,000 bp upstream of the starting codon) are distinguished by shapes and colors. Displayed numbers of varying colors indicate the quantity of each component. Annotations of cis-elements are listed in **Supplementary Table S5**.

The PlantCare online program was utilized to identify putative cis-elements in the 2,000 bp promoter regions of the *PsPIN* genes. A total of 41 cis-regulatory elements were identified and classified into three categories: growth and development, phytohormone, and stress response (**Figure 4**). Growth and development responsive elements primarily included photoresponse (MRE, TCT-motif, AE-box, G-box, Box 4, I-box, GATA-motif, GT1-motif, ACE, TCCC-motif, TGACG-motif, AAAC-motif, Gap-box, CGTCA-motif, GA-motif, L-box and ATCT-motif), zein metabolism regulation (O^2–^site), endosperm expression (GCN4-motif) and meristem expression (GAT-box). Phytohormone responsive elements were comprised of responses to abscisic acid (ABRE), methyl jasmonate (TGACG-motif, CGTCA-motif), gibberellin (P-box, GARE-motif, and TATC-box), auxin (CATATG-site, ARF-binding, ACTTTA-site, TGA-element, and AuxRR-core) and salicylic acid (TCA-element). Elements associated with stress response mainly included responses to wounding (WUN-motif), defense and stress response (TC-rich), stress (STRE), low temperature (LTR, W box), drought (MBS), anaerobic induction (ARE) and other complex stress elements (MYB and MYC). Interestingly, with the exception of *PsPIN2b*, all members of the *PIN* gene family, exhibited various types of auxin -responsive elements.

**Figure 5:**
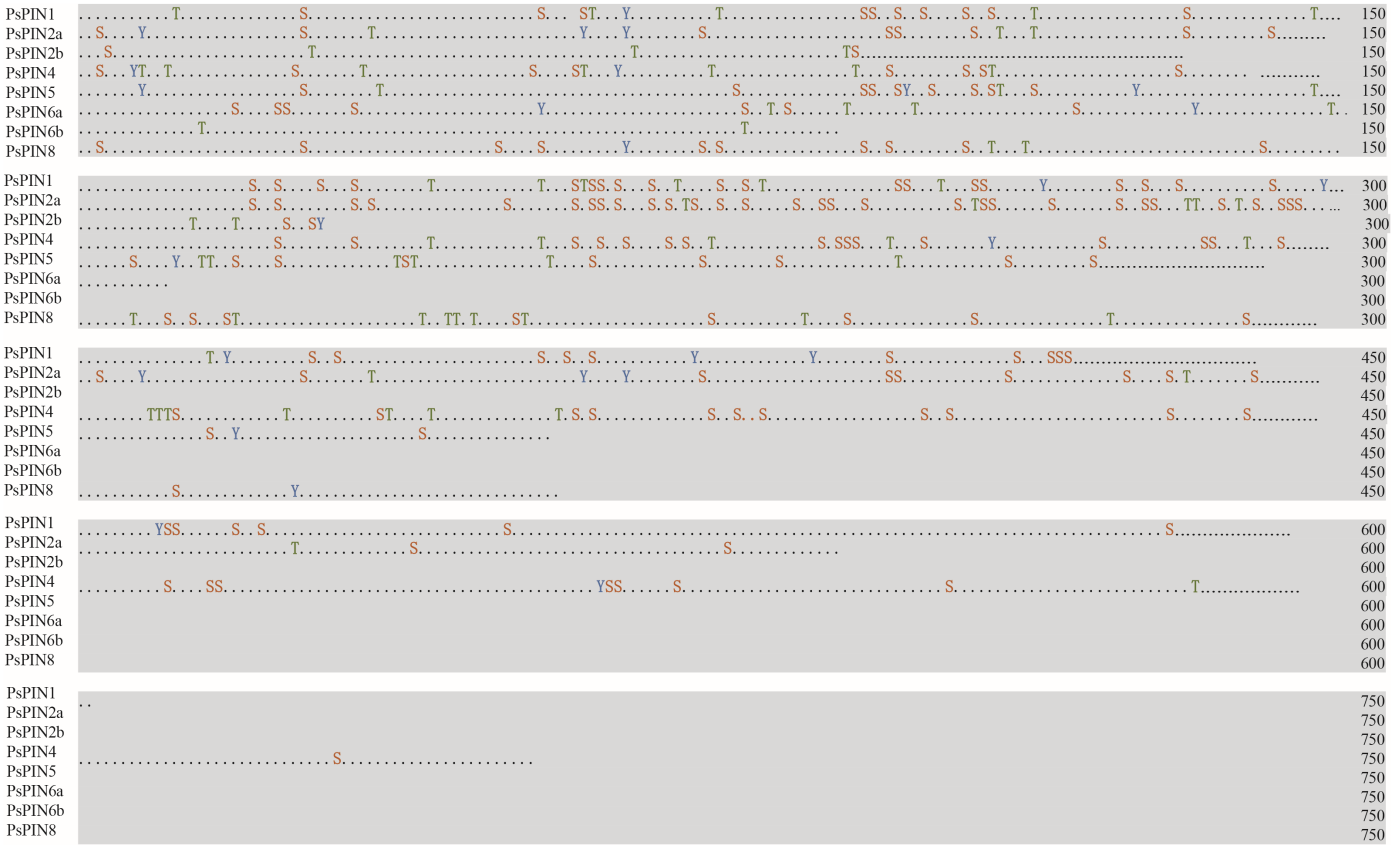
Prediction of PsPIN proteins phosphorylation sites. The serine, threonine and tyrosine are indicated in red, green and blue, respectively. Phosphoric acid sites and kinases are listed in **Supplementary Table S6**.

The online software NetPhos 3.1 Server was used to predict post-translational protein phosphorylation. Phosphorylation sites were abundant among the 8 PsPIN proteins, with serine (Ser) representing the most frequently modified site, followed by threonine (Thr) and tyrosine (Tyr) (**Figure 5**; **Table 2**). The concentration of prediction sites in the center of the hydrophilic ring resulted in significantly fewer sites accounting for PsPIN5 and PsPIN8.

**Table 2:** The predicted phosphorylation sites of PsPIN proteins.

Gene	Serine	Threonine	Tyrosine	Threshold
PsPIN1	45	12	7	64
PsPIN2a	49	11	6	66
PsPIN2b	4	4	1	9
PsPIN4	43	20	5	68
PsPIN5	20	9	5	34
PsPIN6a	7	4	2	13
PsPIN6b	0	2	0	2
PsPIN8	18	10	2	30

### 3.5 Gene ontology and expression patterns analysis of *PsPIN* genes

**Figure 6:**
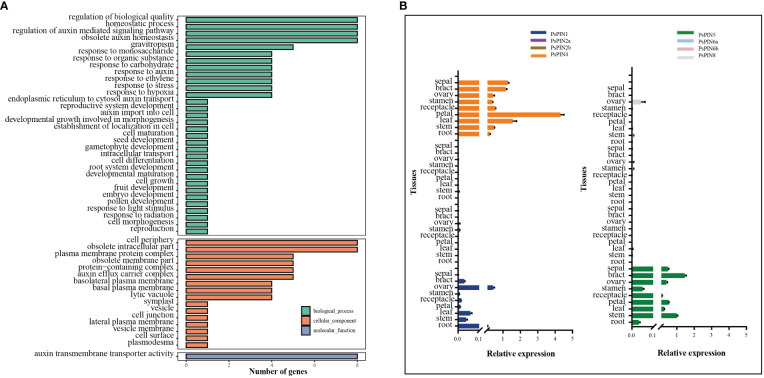
Gene ontology (GO) and expression patterns of *PsPIN* genes. **(A)** GO categories are indicated by green, red, and blue boxes respectively. Annotation details are listed in **Supplementary Table S7**. **(B)** Tissues expression of *PsPIN* genes in the root, stem, leaf, petal, receptacle, stamen, ovary, bract and sepal as determined through qRT-PCR. Error bars are obtained from three measurements.

Gene ontology analysis was performed to elucidate the functional classifications of *PsPIN* genes and explore their involvement in various biological processes (**Figure 6A**). The studied genes were most highly represented in the “Biological Process” ontology by the following GO terms: “regulation of biological quality,” “homeostatic process,” “regulation of auxin-mediated signaling pathway,” and “obsolete auxin homeostasis”. Similarly, genes were prevalent in the “cell periphery” and “obsolete intracellular part” terms of the “Cellular Component” ontology. Notably, the only observed cellular function among the 8 genes was related to auxin transmembrane transport activity. This analysis indicates that *PsPIN* genes are likely involved in various regulatory pathways in plants, including cell morphogenesis, gravitropism, embryo development, and root system development. Furthermore, this analysis revealed new functional categories, such as response to hypoxia, plasmodesma regulation, monosaccharide response, and gametophyte development. Taken together, these findings provide valuable insights for future investigations into *PsPIN* functional divergence.

To better understand the function of *PIN* genes in tree peony, we next assessed the expression of *PsPIN* genes in nine distinct tissues (roots, stems, leaves, petals, receptacles, stamens, ovaries, bracts, and sepals) using qRT-PCR analysis (**Figure 6B**). The result revealed a minimal expression of *PsPIN2* and *PsPIN6* in any tissue and an exclusive expression of *PsPIN8* in the ovary tissue. In contrast, the expression of *PsPIN1*, *PsPIN4*, and *PsPIN5* varied across different tissues. Notably, *PsPIN4* exhibited a significantly high expression in the petal tissue.

### 3.6 Expression patterns of *PsPIN* genes during petal abscission

**Figure 7:**
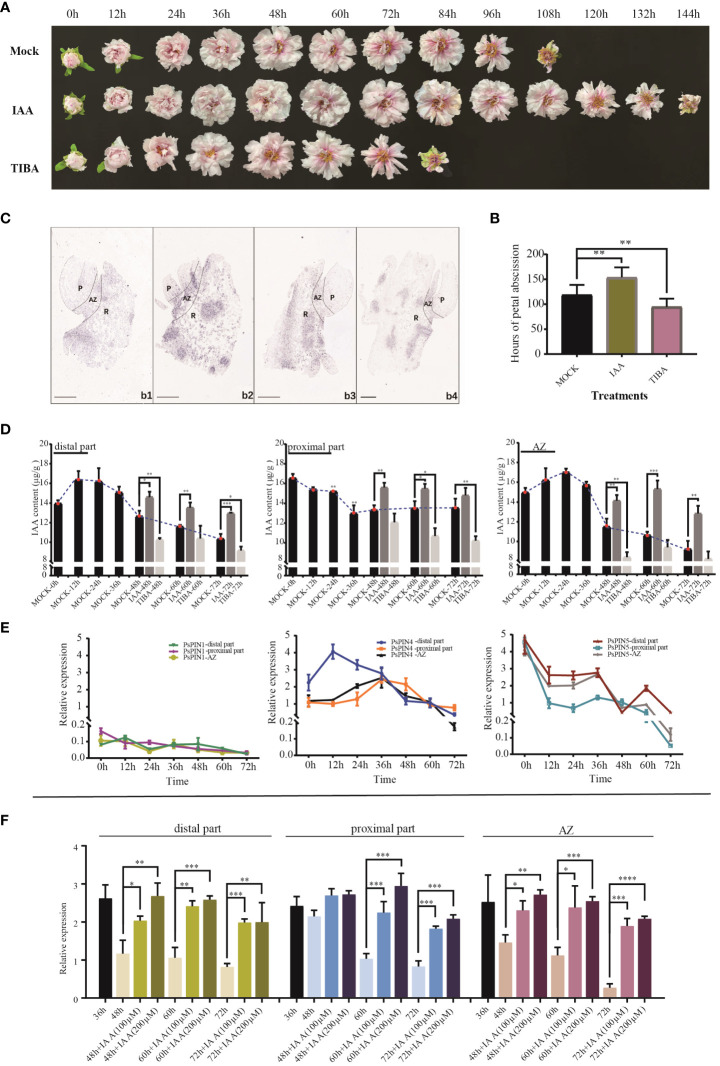
Expression patterns of *PsPIN* genes during petal abscission. **(A)** The flowering process of tree peony under IAA and TIBA treatments. **(B)** Hours of petal shedding following IAA and TIBA treatments. **(C)** *In situ* hybridization of *PsPIN4* in AZ during petal abscission. b1-b4, AZ at 0, 24, 48, and 72 h, respectively. P, Petal; R, Receptacle. Bar=500 μm. **(D)** IAA content in distal part, AZ, and proximal part under IAA and TIBA treatments during petal abscission. **(E)** Relative expression level of *PsPIN1*, *PsPIN4* and *PsPIN5* in distal part, AZ, and proximal part during petal abscission. **(F)** Relative expression level of *PsPIN4* in distal part, AZ, and proximal part by IAA treatment after 36 h. P < 0.05 (∗), P < 0.01 (∗∗), and P < 0.001 (∗∗∗).

Auxin’s influence on the rate of petal shedding in cut peony flowers was examined through the use of IAA and TIBA (**Figure 7A**). The average abscission time following IAA treatment (153.6 h) was 29.30% longer when compared with the control (118.8 h). Conversely, TIBA treatment was found to significantly accelerated the abscission process, resulting in an average abscission time of only 94.8 hours (**Figure 7B**).

The quantification of internal plant IAA following exogenous IAA and TIBA treatments was used to reflect auxin’s influence. Following IAA treatment, there with a significant increase in the distribution of the hormone within the distal part, proximal part, and AZ. After 72 h, IAA levels within the distal portion and the entire AZ were elevated compared to the control, suggesting that shedding had not been initiated. TIBA treatment, however, decreased the quantities of IAA in the three tissues, especially in the distal portion and AZ. Following 48 h, auxin was only detected in very low quantities in both, reaching stabilization in the middle to late flowering period (**Figure 7D**).

AZ initiation is dependents on auxin level changes both internally and in surrounding tissues. However, the crucial functions performed by PIN transporters in this process, particularly in flower organ shedding, have seldom been studied. To screen potential *PsPINs* involved in petal abscission initiation, We analyzed the expression patterns of *PsPIN1*, *PsPIN4*, and *PsPIN5* in various regions (distal part, proximal part, and AZ) during flowering stages (**Figure 7E**). The expression of *PsPIN1* was consistently low across all tissues. In the distal part, *PsPIN4* expression initially increase rapidly at 12 h, followed by a gradual decline. A similar pattern was observed in the proximal part and AZ, with an initial upward trend before 36 h followed by subsequent decline. Notable, the expression of *PsPIN4* in the distal part was lower than that in the proximal part after 36 h. The expression of *PsPIN5* exhibited a generally declining trend across all three tissues, with a significant increase observed at 60 h in the distal part. These findings highlight *PsPIN4* as a candidate gene involved in the regulating of petal abscission.

The application of *in situ* hybridization enabled the visualization of *PsPIN4* expression patterns during petal shedding. The results confirmed the findings of qRT-PCR analysis. Initially, the were only minimal hybridization signals were detected within the AZ, but by 24 h, signals were widespread throughout the entire region (**Figure 7C**). Nevertheless, strong signals persisted on the upper surface of proximal parts, as well as within the vascular bundles and their periphery. By 72 h of flowering, the signals marginally localized within the vascular bundles of the receptacle, reflecting a decline in auxin transport efficiency mediated by PsPIN4. To further investigate the underlying relationship between *PsPIN* and auxin, exogenous IAA was applied 36 hours after flowering and *PsPIN4* expression was analyzed after an additional 48 h (**Figure 7F**). The localized changes in IAA concentration provided feedback on the expression of *PsPIN4*, actively regulating the auxin distribution among tissues. A significant increase in *PsPIN4* expression was observed in the distal part, AZ, and proximal part. Due to continuous IAA supply, *PsPIN4* expression was consistently elevated and did not initiate the abscission layer at 72 h. Furthermore, higher expression levels were more evident with a treatment concentration of 200 μM compared to 100 μM. Overall, our study suggests that *PsPIN4*-mediated auxin transport may be involved in petal abscission.

### 3.7 Ectopic expression of *PsPIN4* promoter and subcellular localization of PsPIN4

**Figure 8:**
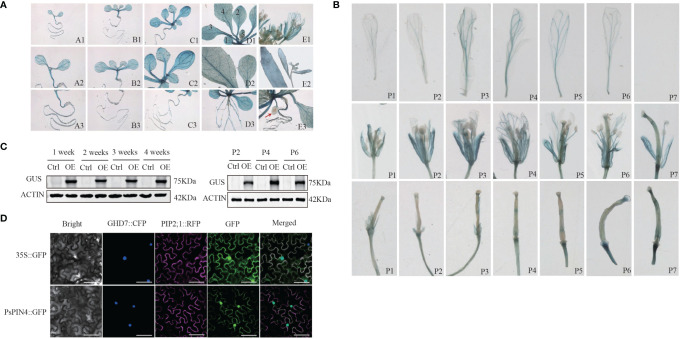
Ectopic expression of *PsPIN4* promoter and subcellular localization of PsPIN4 **(A)** GUS expression driven by *PsPIN4* promoter in *Arabidopsis*. a-e: *Arabidopsis* seedlings at age of 1, 2, 3, 4, and 7 weeks, respectively. Numbers in the d1 represent leaf developmental stages. **(B)** GUS expression is driven by *PsPIN4* promoters in flower organs at different flowering stages. P1~P7: Position of flowers in the inflorescence from top to bottom. **(C)** GUS protein levels assessed by western blot using GUS antibody in plants treated as in **(A)** Actin was detected as a loading control. P2, P4, and P6: Corresponding to the different positions of flower organs in panel **(B)**. **(D)** Subcellular localization of PsPIN4 by fluorescent microscopy with a stimulating wave length of 488 nm, Bar=25 μm.

The ectopic expression of the *PsPIN4* promoter was employed to further elucidate the functional role of PsPIN4. The GUS signal in transgenic *Arabidopsis* was recorded at 1, 2, 3, 4, and 7 weeks respectively. Western blot showed that the GUS fusion protein driven by the *PsPIN4* promoter was effectively expressed in the experimental group (**Figure 8C**). The GUS signal was predominantly distributed in the roots, leaves, petals, sepals and receptacle (**Figure 8A**). In newly formed leaves, the signal was primarily localized to the main vein. As the leaves matured, apart from the main vein, the signal exhibited a punctate distribution throughout the entire area. However, this punctate distribution was less pronounced in aged leaves. Before three weeks of age, the signal spread throughout the entire root and then weakened at its base while becoming concentrated in lateral and apical regions. Our focus of observation encompassed seven positions from top to bottom of the inflorescence for analyzing the signal within flower organs (**Figure 8B**). The signal at the apex of the stigma remained relatively stable during the early and middle stages, but exhibited a significant enhancement at positions 6 and 7. The signal within the petals initially increased and subsequently decreased, with peak intensity observed at position 3 and position 4. This signal was primarily localized along the floral veins. Additionally, there was a gradual signal increase at the junction between petals and receptors, which was particularly evident during early pod development.

The subcellular localization analysis revealed that PsPIN4::GFP exhibited co-localization in both the nucleus and plasma membrane, with a slightly stronger fluorescence signal observed in the nucleus compared to the plasma membrane (**Figure 8D**).

### 3.8 Ectopic expression of *PsPIN4* in *Arabidopsis* delays floral organ abscission

**Figure 9:**
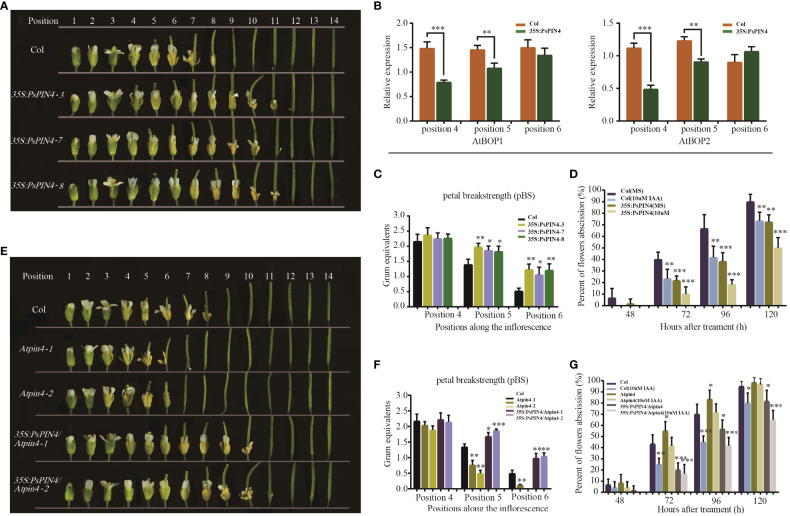
Ectopic expression of *PsPIN4* in *Arabidopsis* delays floral organ abscission. **(A)** Floral organ abscission in the Col and 35S:PsPIN4 transgenic lines. Numbers indicate floral positions along the inflorescence. **(B)** qRT-PCR analysis of *AtBOP1* and *AtBOP2* expression in Col and transgenic lines. **(C)** Petal breakstrength (pBS) of Col and transgenic lines (n=20, bars=SD). **(D)** Floral organ abscission process of Col and *35S:PsPIN4* under IAA treatment. Data were collected every 24 h starting from 48 h. **(E)** Floral organ abscission in Col, *Atpin4* mutant and *35S:PsPIN4/Atpin4* lines. **(F)** pBS of Col, *Atpin4* mutant and *35S:PsPIN4/Atpin4* transgenic lines. (n=20, bars=SD). **(G)** Floral organ abscission rate of Col, *Atpin4* mutant and *35S:PsPIN4/Atpin4* transgenic lines under IAA treatment. Stars indicate significant pairwise differences according to the T-test. *P < 0.05, **P < 0.01, ***P < 0.001, ****P < 0.0001.

To further investigate the functions of *PsPIN4*, we examined its impact on floral organ shedding in *Arabidopsis*. In wild-type Col *Arabidopsis*, the floral organs began to fully drop at position 8. Conversely, transgenic *Arabidopsis* lines overexpressing *PsPIN4* (*35S:PsPIN4-3*, *35S:PsPIN4-7*, and *35S:PsPIN4-8*) exhibited a significant delay in petal abscission time, with floral organs fully dropping at position 12 or 13 (**Figure 9A**). *BLADE-ON-PETIOLE* (*BOP*) was used as a gene marker for AZ formation (McKim et al., 2008). The expression of *AtBOP1* and *AtBOP2* were significantly reduced at position 4 and 5 in *35S:PsPIN4* compared to Col (**Figure 9B**). Furthermore, the delay in floral organ abscission was quantified by measuring petal break strength (pBS) (**Figure 9C**). The transgenic lines required more force than Col to tear petals from flowers at positions 5 and 6. Additionally, flowers with “just-visible” white petals were selected and cultured on IAA plates to record the abscission rate (**Figure 9D**). Initially, the abscission process was found to be significantly delayed in Col plants grown on IAA plates compared to MS plates. However, abscission rates for transgenic lines grown on MS plates and Col grown on IAA plates were similar. Additionally, the *35S:PsPIN4* cultures on the IAA plate demonstrated prolonged organ abscission when compared to all other groups, resulting in only a 47.93% abscission rate at 120 h. While further examining the abscission process of *Atpin4*, we observed premature abscission of flower organs, with dropping occurring at positions 7 or 8 (**Figure 9E**). Interestingly, the introduction of *PsPIN4* under the control of the CaMV35S promoter into the *Atpin4* background seemed to rescue this phenotype, as floral organs completely detached at positions 11 or 12 with a dropout rate similar to that of *35S:PsPIN4* (**Figure 9A**). The pBS measurement and abscission rate of the IAA-treated plate further confirmed these results (**Figure 9F**). Moreover, the abscission rates of the *Atpin4* and Col groups on the IAA plate were similar at 120 h, while the *35S:PsPIN4/Atpin4* groups displayed significantly lower abscission rates than the other groups (**Figure 9G**). The pBS at positions 5 and 6 also indicated that the repair line could effectively delay petal shedding (**Figures 9E, F**). Collectively, this data strongly supports the role of *PsPIN4* in delaying floral organ abscission in *Arabidopsis*.

## 4 Discussion

The number of *PIN* genes varies widely among plants. We identified 8 *PIN* genes in tree peony, as many as *Arabidopsis*, but lower than several other plant species, such as rice, cotton, maize, and soybean, which have 13, 11, 12, and 23 *PIN* genes, respectively. The genome size of tree peony is about 12.28 Gb (Yuan et al., 2022), while that of *Arabidopsis*, rice, maize, and soybean is 125 Mb, 430 Mb, 2300 Mb, and 1.025Gb, respectively (Arabidopsis Genome, 2000; Burr, 2002; Schnable et al., 2009; Schmutz et al., 2010; Sabot et al., 2011). In this case, it appears that there is no direct correlation between the number of *PIN* genes and genome size in these plants. The analysis of 20 candidate motifs revealed that the PIN family can be classified into three distinct categories. The “long” PIN class predominantly consists of 20 motifs, whereas the “short” PIN class lacks motifs in the central hydrophilic ring region. Although the motifs found in PsPIN members exhibit variation, they still adhere to this classification scheme. The absence of multiple motifs in PsPIN2b, PsPIN6a and PsPIN6b could be attributed to genome annotation. The structural composition of the central hydrophilic region of PIN proteins essential for classification within this protein family. The modification of this region can result in subfunctionalization through selective deletion or repetition (Zhou and Luo, 2018). Multiple sequence alignment analyses revealed that PsPIN1, PsPIN2a and PsPIN4 share sequences with an identity exceeding 60% in both the central hydrophilic region and each structural domain. The hydrophilic regions of typical PIN proteins have the potential to facilitate specific protein-protein interactions in eukaryotes (Krecek et al., 2009). Conserved PsPIN family proteins may contribute to protein localization overlap and functional synergy, redundancy, or antagonism (Rakusova et al., 2015). For instance, AtPIN3, AtPIN4 and AtPIN7 exhibit compensatory functions in regulating phototropism growth and embryonic development in Arabidopsis (Friml et al., 2003; Paponov et al., 2005), thus, highly conserved PsPIN proteins may also demonstrate similar characteristics.

PIN protein, play an important role in intricate regulatory networks within plants auxin signaling as crucial transporter (Friml, 2003). The auxin response of the *PIN* gene is influenced by both tissue type and developmental stage, which may explain the diverse auxin elements observed in *PsPINs* promoters. Regulation of PIN by certain hormones also contributes to its interaction with auxin or other hormones. Auxin has been reported to facilitate the degradation of DELLA proteins, thereby enhancing cellular response to gibberellin (GA) (Fu and Harberd, 2003). Conversely, GA promotes localization of the PIN protein on the cell membrane while inhibiting auxin transport (Willige et al., 2011; Loefke et al., 2013). Additionally, treatment with Jasmonic acid (JA) down-regulates expression levels of *AtPIN7* leading to a deceleration in auxin transport (Jang et al., 2019). The abundance of hormone-responsive elements in the *PsPIN* promoters suggests that this protein family interacts with a variety of other hormones. Specific phosphorylation modifications play a crucial role in determining the polar orientation and transport function of PIN proteins (Huang et al., 2010). PsPIN proteins have multiple modification sites primarily within the region spanning 200-300 amino acids. However, further research is needed to describe the regulatory pathway governing PIN phosphorylation during specific growth and development stages. Conserved loci are typically found within TPRXS motifs present in long hydrophilic rings (Zourelidou et al., 2014; Dory et al., 2018). In *Arabidopsis*, AtPIN8 protein lacking phosphorylation sites within the short ring domain exhibits independent transport activity from kinases (Mounet et al., 2012). The “short” PIN type of PsPIN5 and PsPIN8 correlates to a significant reduction in serine and threonine modification sites, thereby providing further support for this perspective.

The expression of *PIN* genes is regulated by auxin through the Aux/IAA signal transduction system, and their auxin response varies depending on tissue type (Heisler et al., 2005; Gallavotti et al., 2008; Bayer et al., 2009). In *Arabidopsis*, auxin treatment induces the expression of *AtPIN1*, *AtPIN3*, and *AtPIN7* in roots but not in the hypocotyl (Vieten et al., 2005). During shedding, the *PsPIN4* gene exhibited a heightened sensitivity to IAA at both the proximal and distal parts. Following the absorption and accumulation of exogenous IAA in the receptacle, local concentration changes were perceived by the tissues, resulting in an upregulation of *PsPIN4* expression. Consequently, auxin was transported to the petals through junctions to facilitate flowering, inducing an upregulation of *PsPIN4* expression at the distal part. Additionally, *PsPIN4* expression depends on IAA concentration as a means to cope with changes in plant auxin levels and actively regulate internal homeostasis. However, regulation of *PIN* by auxin is complex as prolonged exposure or high concentrations can promote *PIN* entry into cells resulting in degradation (Sieberer et al., 2000; Vieten et al., 2005). Therefore, further investigation is required to uncover the relationship between PsPIN4 and auxin distribution during the dissociation stage of the organ shedding process. The IAA concentrations of both the proximal and distal parts undergo dynamic changes during organ shedding (Taylor and Whitelaw, 2001). The level of the distal part is consistently decreased to facilitate detachment (Kucko et al., 2020). This study revealed that during the middle and late stages of flowering, *PsPIN4* expression at the distal part continued to decline as a response to auxin transport efficiency in preparation for abscission. Furthermore, it appears that the rate of auxin transport in the AZ may have a more direct impact on the abscission process than simply relying on concentration differences between both sides (Liang et al., 2020). During early flowering stages, a significant accumulation of *PsPIN4* occurred within and around the AZ to facilitate IAA transport for meeting flowering. However, its distribution within AZ significantly diminished later on, indicating a weakened capacity for IAA transport. The distribution pattern of *PsPIN4* observed during late receptacle stages may be attributed to either the partial auxin supply required for protective layer formation post-abscission or the response of the fractured layer to external exposure.

Previous studies have demonstrated significant differences in the expression of various *PINs* (Habets and Offringa, 2014). The ectopic expression of the promoter suggests that *PsPIN4* might not only be involved in flowering regulation, but may also assist in the development of roots, leaves, and fruits Furthermore, our analysis of GUS signal distribution characteristics suggest the gene may be active across multiple tissues during flowering. The expression levels observed in petals during early stages followed by continuous decline aligned with expression trends observed in tree peony. Notably, the junction between the receptacle and petal in *Arabidopsis*, corresponded to the fruit pod growth site where sustained high expression signals were crucial for adequate auxin supply to facilitate fruit pod development. This potentially explains inconsistencies observed in *PsPIN4* expression during the late flowering stage of tree peony.

The application of exogenous IAA was found to significantly delay petal abscission in both peony and *Arabidopsis* plants. The overexpression of the *PsPIN4* gene efficiently facilitated IAA transport in the IAA plate, thus sustaining flower presence. However, limitation in the transporter PIN or insufficient IAA content resulted in similar shedding rates following 120 h of *35S:PsPIN4* growth on the MS plate and Col growth on the IAA plate. The premature abscission phenotype observed in mutants suggests the involvement of *AtPIN4* in flower organ abscission. The weakening displayed in IAA plates indicate a potential participation by functionally redundant members, requiring further investigation. Our analysis also demonstrated that *35S:PsPIN4/AtPIN4* could effectively compensate for the mutant’s early shedding phenotype. Overall, these findings indicate that PIN4-mediated auxin transport plays a crucial role in *Arabidopsis* flower organ abscission.

In summary, our study revealed a total of 8 *PsPIN* genes were identified from the tree peony genome, which could be classified into “long” and “short” PIN categories depending on their structure. These genes were randomly distributed across three tree peony chromosomes. With the exception of PsPIN2b, PsPIN6a, and PsPIN6b (due to incomplete genome annotation), PsPIN protein sequences exhibited a high degree of conservation, which was characterized by the presence of 4-5 transmembrane domains at both the N-terminal and C-terminal regions. *PsPINs* promoters were found to contain cis-regulatory elements associated with growth, development, phytohormone responsiveness, and environmental stress. Furthermore, we examined the differential expression patterns of *PsPIN* genes in various tissues. The presence of diverse phosphorylation sites in the hydrophilic region suggests complex post-translational modification mechanisms, which will require further study. Perhaps most importantly, we discovered that *PsPIN4* is highly sensitive to auxin in abscission zones and acts as a floral organ abscission repressor. These findings provide valuable insights for future *PsPIN* gene investigations and contributes to our understanding of the intricate relationship between PIN-mediated auxin transport and abscission.

## Data availability statement

The datasets presented in this study can be found in online repositories. The names of the repository/repositories and accession number(s) can be found in the article/**Supplementary Material**.

## Ethics statement

The experiments did not involve endangered or protected species. The data collection of plants was carried out with permission of related institution, and complied with national or international guidelines and legislation.

## Author contributions

YS: Writing – original draft, Writing – review & editing, Conceptualization, Data curation, Formal Analysis, Software, Supervision, Validation, Visualization. JC: Conceptualization, Data curation, Writing – original draft. YY: Investigation, Software, Writing – review & editing. NJ: Methodology, Project administration, Writing – original draft. CL: Funding acquisition, Methodology, Validation, Writing – original draft. YZ: Data curation, Investigation, Supervision, Writing – original draft. XM: Formal Analysis, Project administration, Visualization, Writing – review & editing. QZ: Data curation, Formal Analysis, Visualization, Writing – review & editing. YF: Conceptualization, Formal Analysis, Methodology, Supervision, Writing – original draft. ZS: Formal Analysis, Funding acquisition, Project administration, Resources, Writing – review & editing. **SG:** Conceptualization, Funding acquisition, Project administration, Resources, Supervision, Writing – review & editing.
