# Supplementary material for: Auxin efflux carrier PsPIN4 identified through genome-wide analysis as vital factor of petal abscission
Source: Front Plant Sci. 2024 May 10;15:1380417. doi: 10.3389/fpls.2024.1380417 (PMC11116700; doi:10.3389/fpls.2024.1380417)
Supplement: Supplementary file 1 [file DataSheet_1.zip › Supplementary Materials/Table S3 Sequences of 20 predicted motifs of PsPIN proteins.docx]

**Table S3** **Sequences of 20 predicted motifs of PsPIN proteins**

| **Motif** | **Width** | **Motif Sequence** |
| --- | --- | --- |
| 1 | 50 | PLYVAMILAYGSVKWWKIFTPDQCSGINRFVALFAVPLLSFHFISTNBPY |
| 2 | 29 | AIVQAALPQGIVPFVFAKEYNVHPDILST |
| 3 | 29 | LEWSITLFSLSTLPNTLVMGIPLLKAMYG |
| 4 | 39 | MPAIIAK29SISILSDAGLGMAMFSLGLFMALQPKIIAC |
| 5 | 29 | GSLMVQIVVLQCIIWYTLLLFLFEYRGAK |
| 6 | 29 | MVWRKLIRNPNTYSSLJGLIWSLISFRWN |
| 7 | 29 | AMAVRFLTGPAVMAAASIAVGLRGVLLHV |
| 8 | 21 | AVIFGMLIALPITLVYYILLG |
| 9 | 30 | SSMTPRPSNLTGAEIYSLQSSRNPTPRGSS |
| 10 | 21 | AMNLRFIAADTLQKLJVLVVL |
| 11 | 23 | ZTEAEVGDDGKLHVTVRKSNASR |
| 12 | 28 | GKAAAKGNHDGKDLHMFVWSSSASPVSE |
| 13 | 15 | MITWKDLYHVLTAVV |
| 14 | 15 | KQMPPASVMTRLILI |
| 15 | 15 | SFKVDSDVVSLDGRE |
| 16 | 15 | LLIMEQFPDTAASIV |
| 17 | 15 | SRGPTPRPSNFEEDG |
| 18 | 15 | FNHTDFYSMMGGGGN |
| 19 | 11 | ALWANFSKRGS |
| 20 | 15 | DEDYFERDEFSFGNR |
