# Supplementary material for: Auxin efflux carrier PsPIN4 identified through genome-wide analysis as vital factor of petal abscission
Source: Front Plant Sci. 2024 May 10;15:1380417. doi: 10.3389/fpls.2024.1380417 (PMC11116700; doi:10.3389/fpls.2024.1380417)
Supplement: Supplementary file 1 [file DataSheet_1.zip › Supplementary Materials/Table S8 Primers .docx]

**Table S8 Primers**

| Primer Name | Forward Primer (5′→3′) Sequence | Reverse Primer (5′→3′) Sequence |
| --- | --- | --- |
| PsActin | ACCTCTATGGCAACATTGTGCTCAG | CTGGGAGCCAAAGCGGTGATT |
| qRT-PsPIN1 | CGTGGCCATGATCCTAGCTT | GAGAACCCCTTGAGCTGACC |
| qRT-PsPIN2a | AAACGATGAGTCCGATGCGA | CGCAGGCTATGAGTTTTGGC |
| qRT-PsPIN2b | CACCCACCTAAGCTCCGTTC | TGCAGTACGGTATCGGTTTCG |
| qRT-PsPIN4 | GGAAACGACGGGAAGCTACA | TACAAATCCGCCGGACCAAA |
| qRT-PsPIN6a | TGCCATTGTCCTTTGTGGGG | CCAAAACGGGCGCAGTAGAA |
| qRT-PsPIN6b | AATTGGCCTTTGCAGGGTTC | TGGGCAACAGTACCAACATTC |
| qRT-PsPIN5 | ACGGGGACTGCCATGTTTAG | CCAAGACCGGAAGAGACACC |
| qRT-PsPIN8 | GGAGCTGGAGAACCCACAAG | CGGCAATTCTACTCCCCACC |
| PsPIN4-GUS | ACGAATTCCCGGGGATCCGTCGACATATCTTTTTGCCACGGTTATAC | GTAAAACGACGGCCAGTGCCAAGCTTTTTATTTGAAGTCTGAAAAAATTC |
| 35S:PsPIN4 | ATCTCGATACACCAAATCGACTCTAGAATGATTTCTTGGCAT | GCTCCTCGCCCTTGCTCACCATGGTACCCA ATCCAAGAACAA |
| AtBOP1 | ACGAAGAGGAGCAAAGGAGC | ATCGGAGTCGAAATCGTCGG |
| AtBOP2 | GTGGGACAGACTCACCACAA | TCTAGGCTCGTGTTTCTGCG |
| PsPIN4-GFP | ATCTCGATACACCAAATCGACTCTAGAATGATTTCTTGGCAT | GCTCCTCGCCCTTGCTCACCATGGTACCCA ATCCAAGAACAA |
