# Supplementary material for: Auxin efflux carrier PsPIN4 identified through genome-wide analysis as vital factor of petal abscission
Source: Front Plant Sci. 2024 May 10;15:1380417. doi: 10.3389/fpls.2024.1380417 (PMC11116700; doi:10.3389/fpls.2024.1380417)
Supplement: Supplementary file 1 [file DataSheet_1.zip › Supplementary Materials/Table S9 Probe sequence in situ hybridization .docx]

**Table S9 Probe sequence In situ hybridization**

| **Name** | **Width** | **Sequence** |
| --- | --- | --- |
| Antisense probe | 156 | ACGTGGTTGTTTGAGGTGGTGGTGGTGGCGGCGGCGGTGCGCAATTTTCCTCGAAATTCGACGGCCTTGGCGTTGGACCCCTTGATGACTGAACAGAATACAAATCCGCCGGACCAAAATTAGAGTGCCTCGCCGGGAACCCTTGGCATCCCATCA |
